# Feasibility of Quantitative Magnetic Resonance Fingerprinting in Ovarian Tumors for T_1_ and T_2_ Mapping in a PET/MR Setting

**DOI:** 10.1109/TRPMS.2019.2905366

**Published:** 2019-03-15

**Authors:** Joshua D. Kaggie, Surrin Deen, Dimitri A. Kessler, Mary A. McLean, Guido Buonincontri, Rolf F. Schulte, Helen Addley, Evis Sala, James Brenton, Martin J. Graves, Ferdia A. Gallagher

**Affiliations:** Department of Radiology, University of Cambridge, Cambridge CB2 0QQ, U.K.; Cambridge University Hospitals, NHS Foundation Trust, Addenbrooke’s Hospital, Cambridge, U.K.; Department of Radiology, University of Cambridge, Cambridge CB2 0QQ, U.K.; Cambridge University Hospitals, NHS Foundation Trust, Addenbrooke’s Hospital, Cambridge, U.K.; Department of Radiology, University of Cambridge, Cambridge CB2 0QQ, U.K.; Cambridge University Hospitals, NHS Foundation Trust, Addenbrooke’s Hospital, Cambridge, U.K.; Cancer Research U.K. Cambridge Institute, University of Cambridge, Cambridge CB2 0RE, U.K.; IMAGO7 Foundation, 56128 Pisa, Italy; GE Healthcare, D-80807 Munich, Germany; Department of Radiology, University of Cambridge, Cambridge CB2 0QQ, U.K.; Cambridge University Hospitals, NHS Foundation Trust, Addenbrooke’s Hospital, Cambridge, U.K.; Department of Radiology, University of Cambridge, Cambridge CB2 0QQ, U.K.; Cambridge University Hospitals, NHS Foundation Trust, Addenbrooke’s Hospital, Cambridge, U.K.; Cancer Research U.K. Cambridge Institute, Cambridge CB2 0RE, U.K.; Cancer Research U.K. Cambridge Institute, University of Cambridge, Cambridge CB2 0RE, U.K.; Department of Radiology, University of Cambridge, Cambridge CB2 0QQ, U.K.; Cambridge University Hospitals, NHS Foundation Trust, Addenbrooke’s Hospital, Cambridge, U.K.; Department of Radiology, University of Cambridge, Cambridge CB2 0QQ, U.K.; Cambridge University Hospitals, NHS Foundation Trust, Addenbrooke’s Hospital, Cambridge, U.K.

**Keywords:** Cancer applications, clinical imaging, imaging techniques, magnetic resonance imaging (MRI), oncology, ovarian cancer

## Abstract

Multiparametric magnetic resonance imaging (MRI) can be used to characterize many cancer subtypes including ovarian cancer. Quantitative mapping of MRI relaxation values, such as *T*_1_ and *T*_2_ mapping, is promising for improving tumor assessment beyond conventional qualitative *T*_1_- and *T*_2_-weighted images. However, quantitative MRI relaxation mapping methods often involve long scan times due to sequentially measuring many parameters. Magnetic resonance fingerprinting (MRF) is a new method that enables fast quantitative MRI by exploiting the transient signals caused by the variation of pseudorandom sequence parameters. These transient signals are then matched to a simulated dictionary of *T*_1_ and *T*_2_ values to create quantitative maps. The ability of MRF to simultaneously measure multiple parameters, could represent a new approach to characterizing cancer and assessing treatment response. This feasibility study investigates MRF for simultaneous *T*_1_, *T*_2_, and relative proton density (rPD) mapping using ovarian cancer as a model system.

## I Introduction

OVARIAN Cancer is the second leading cause of death from gynaecologic cancers [1]. Imaging methods, including magnetic resonance imaging (MRI), positron emission tomography (PET), and SPECT seek to improve the identification of tumor subtypes as well as their response to treatment. Current clinical MRI methods cannot characterize all tumor subtypes, and therefore better methods must be found in order to create more specific disease biomarkers.

Clinical MR imaging acquires multiple images with different approaches to generate contrast and are usually assessed qualitatively. Contrast on MR imaging is based on differences in magnetic resonance parameters in tissue, such as longitudinal *T*_1_ relaxation, transverse *T*_2_ relaxation, and relative proton density (rPD). Multiple images with different weightings are obtained by varying the acquisition, including parameters, such as repetition time (TR) and flip angle (FA). Although the contrast that is generated is qualitative, contrast is highly dependent on operator specifications, which can complicate the interpretation at multiple centers. In order for the results to be more repeatable and representative of the underlying biological factors that control signal, quantitative MR mapping is essential.

Quantitative mapping of MRI relaxation values is promising for improving tumor diagnosis, for monitoring of disease progression, and for assessment of treatment response beyond simple qualitative assessments [2], [3]. However, traditional quantitative imaging can be inefficient, requiring multiple serial acquisitions from which a single quantitative map can be derived [4]. The measurement of multiple MR parameters is almost always time-consuming and is particularly challenging in moving regions such as the abdomen.

Magnetic resonance fingerprinting (MRF) [4] has recently been introduced, as a novel acquisition and reconstruction strategy to overcome these challenges and time-constraints, with the potential to be used for clinical imaging. MRF could improve the speed and accuracy of PET/MRI parameter quantitation for cancer imaging [5]. MRF in prostate, abdomen, and brain cancer has shown nearly double the *T*_1_ values when compared to normal-appearing tissue, and *T*_2_ differences as large as 70% have been demonstrated between low and high grade tumors [6]–[8].

MRF enables fast, simultaneous, and efficient multiparametric mapping by exploiting the transient signals produced from the variation of pseudo-random sequence parameters. These generated transient signal evolutions or “fingerprints” are unique for different tissues and are dependent on the various magnetic resonance properties of the tissue. After data acquisition, the signals are matched to a simulated dictionary including (but not limited to) a range of *T*_1_ and *T*_2_ values to create quantitative maps. The rPD is the scaling factor used to match the simulated signal evolution with the measured signal.

The aim of this paper was to assess the feasibility of using MRF for simultaneous *T*_1_, *T*_2_, and rPD mapping of ovarian cancer for the first time. We investigated if the addition of multiparametric MRF to conventional MRI measurements could provide information for the characterization of ovarian masses.

## II Materials and Methods

### A Phantom Measurements

MRF and standard relaxation mapping data were obtained from the ISMRM/NIST phantom [9] to assess the accuracy of *T*_1_ and *T*_2_ measurements. Phantom data were obtained on a 3.0 T PET/MR (GE Healthcare, Waukesha, WI, USA) and a standard 3.0 T MR system (MR750, GE Healthcare, Waukesha, WI, USA) using single-channel body coils. Regions-of-interest (ROI) were created from the vials in either the *T*_1_ or *T*_2_ slice of the phantom.

*1) Standard T_1_ Mapping:* Variable FA (VFA) *T*_1_ maps were obtained with a 3-D fast gradient echo sequence with: matrix = 192 × 192 × 72, field-of-view (FOV) = 380 × 380 × 216 mm^3^, FAs = 2°, 5°, 8°, 12°, 15°, 20°, TR = 4.96 ms, echo time (TE) = 2.1 ms, and bandwidth= ±94 kHz. The *T*_1_ maps were created using linear least-squares fit of the obtained signals using the DESPOT1 method [10], using DICOM images and custom MATLAB code (MathWorks Foundation, Massachusetts, USA).

*2) Standard T_2_ Mapping: T*_2_ maps were obtained with a multislice 2-D multiple echo spin echo (MESE) sequence using the following parameters: matrix = 192 × 192 × 21, FOV = 380 × 380 × 168 mm^3^, matrix = 192 × 192 × 21, slice thickness = 3 mm, slice spacing = 5 mm, FA = 90°, TR = 1500 ms, TEs = 7, 14, 21, 28, 35, 42, 49, 56 ms, and bandwidth = ±47 kHz. The *T*_2_ maps were created using a log-linear-least-squares fit with custom MATLAB code.

*3) MRF T_1_ and T_2_ Mapping:* ISMRM/NIST phantom experiments were used to validate MRF on the PET/MR and on the standard MRI system. MRF was repeated on the standard system with follow-ups after an hour and a day, and with three frame lengths to measure these effects on *T*_1_ and *T*_2_ measurements.

**Fig. 1:**
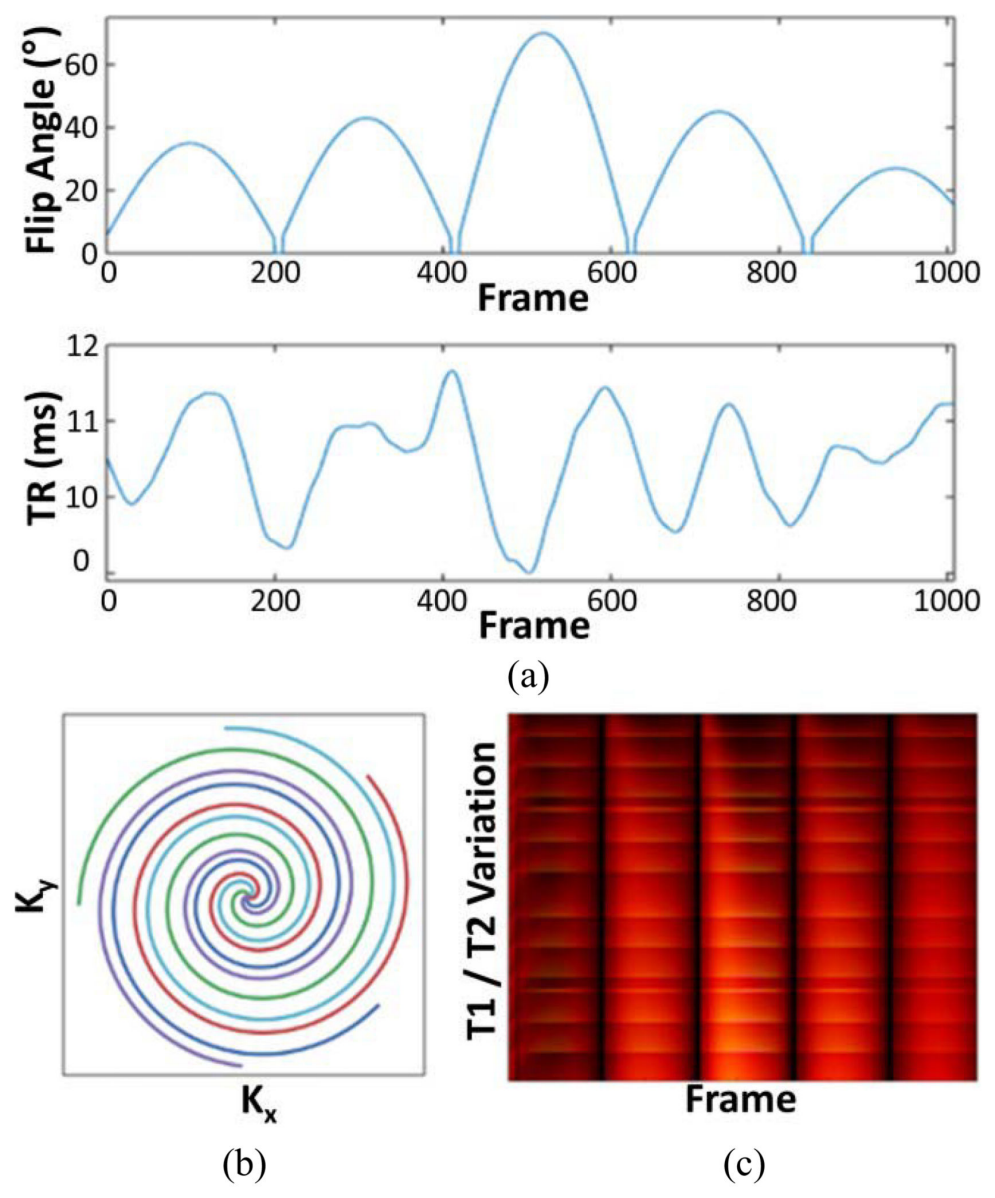
(a) FA and TR list used during acquisition and simulation. (b) Five spokes showing the *k*-space acquisition with golden angle spiral interleaves. (c) Sample dictionary of simulated signals showing the intensity of the MRF signal for each *k*-space spiral acquisition (frame), for different TRs and FAs.

A baseline phantom measurement was performed on the standard 3.0 T system, using an initial inversion pulse, and a 38 cm FOV, like the *in vivo* MRF. MRF acquisitions included baseline (=979), 489 (≈ 1*/*2 * 979) or 244 (≈ 1*/*2 * 979) frames with the initial values of the lists [9], [11] in Fig. 1(b) to assess the effect of frame reduction on mapping accuracy. The baseline scan was repeated after an hour and after a week. The same baseline acquisition was obtained using the PET/MR. The temperature of the standard system was 18° for baseline, 17° for one-week follow-up, and 19° for the PET/MR.

In order to find the optimal rank for singular value decomposition (SVD) compression [12] of the dictionary, the data were reconstructed with ranks between 1 and 20. An optimal SVD rank was determined when the *T*_1_ and *T*_2_ difference was less than 1% with the values at SVD rank ≥ 16.

### B In Vivo Imaging

Patient images were acquired on a standard 3.0 T MRI system using a 32-channel abdominal coil. All patients were imaged in the supine position. The MRI protocol consisted of standard qualitative clinical sequences followed by a 2-D steady-state-free precession (SSFP) MRF sequence [4].

*In vivo* MRF data were acquired using 979 frames with spiral *k*-space interleafs [Fig. 1(a) and (b)]. Each spiral was rotated by the golden-angle for each of 89 interleafs, following which the 89 spirals were then repeated 11 times to fill 979 frames. The golden-angle interleafs enabled the TR and FA lists used to match the values in [9] and [11]. A slice-selective inversion pulse was used prior to FA and TR list variation. Further sequence parameters were: FOV = 380 × 380 mm^2^; matrix = 192 × 192; voxel size = 2.0 × 2.0 × 3.0 mm^3^; slice thickness = 3.0 mm; slice spacing = 1.0 mm; sampling bandwidth = ±250 kHz; TE = 1.8 ms; and acquisition time = 15 s/slice, with 18–22 slices per patient, for a total scan time between 4.5 and 5.5 min. The maximum gradient strength per spiral was 17 mT/m and the maximum slew rate was 62 T/m/s. The gradient strength was limited to low values to ensure that a large FOV would be obtained to cover the whole abdomen.

*T*_1_ and *T*_2_ values for the three tumor types were calculated from ROIs drawn on four consecutive slices. Mean relaxation values for the post-treatment subject were determined from ROIs on eight consecutive slices.

### C MRF Dictionary Simulation

Dictionary simulations [Fig. 1(c)] of the signal evolution from an SSFP acquisition scheme were performed using the extended phase graph formalism [5]. The ranges and incremental (step-size) changes of the *T*_1_ and *T*_2_ values that were included in the dictionary are listed in Table I. The range was chosen to represent both short relaxation times, which occur in dense and fatty tissue, and high relaxation times, which occur in fluid regions such as ascites. The increments were chosen to be smaller near short relaxation times, where the increments are a larger percentage change for each dictionary value.

Three temporally adjacent dictionary values were summed for the final dictionary creation, such that a three-frame sliding window reconstruction and matching technique could be used [13]. This process is possible due to the linearity property of the Fourier transformation, which preserves information under linear transformation. The sliding window increased the signal-to-noise and reduced the undersampling artifact from each frame, which was necessary for accurate matching.

### D MRF Reconstruction

MRF data were collected using an under-sampled spiral acquisition that resulted in 979 under-sampled images per slice. The MRF data were reconstructed using a regridding algorithm to an interpolated Cartesian *k*-space before a fast Fourier transfer (FFT), and used a three frame sliding window [13]. Image reconstruction and dictionary matching was parallelized to increase reconstruction efficiency, which was performed on 48 CPU cores using 400 gigabytes of RAM. After reconstruction of the images from each coil channel, the channels were combined using adaptive coil combination on the average of the time frames [14].

MRF uses a relatively simple pattern recognition algorithm to identify the tissue and its corresponding properties in each voxel. The inner products between the normalized measured signal evolution of each voxel and each normalized dictionary entry are calculated. The dictionary entry returning the maximum value of the inner product is taken as the best representation of the acquired signal evolution. The respective *T*_1_ and *T*_2_ values are consequently assigned to the voxel. The rPD is calculated as the scaling factor used to match the dictionary simulation with the measured signal evolution.

### E SVD Compression

MRF benefits from dimensionality reduction techniques that reduce both the size of the dictionary and of the stored images. Dimensionality reduction enables easier long term storage and faster computation for dictionary matching [12]. SVD is a dimensionality reduction method (or a “principal component analysis” method) that seeks to reduce the number of signal features. SVD performs a linear mapping of the data to a lower dimensional space while maximizing the variance of the reduced set of features. For MRF, both the dictionary and undersampled images are reduced (“compressed” or “factored”), using the SVD decomposition weights that were determined during dictionary compression [12].

Multiple singular vector lengths (or “ranks”) were investigated by varying the rank between 1 and 20 for the phantom measurements in order to investigate the variability that is introduced with the SVD compression. For the *in vivo* measurements, rank 16 was used based on the phantom measurement error being lower than 1%.

### F *In Vivo* Imaging

Four patients were imaged: one borderline serous tumor and three high-grade serous ovarian cancers (HGSOCs). One HGSOC had extensive ascites, and one HGSOC was imaged post chemotherapy with no histological evidence of remaining cancer. All data were acquired with informed consent and local ethical approval.

## III Results

### A Phantom

**Fig. 2:**
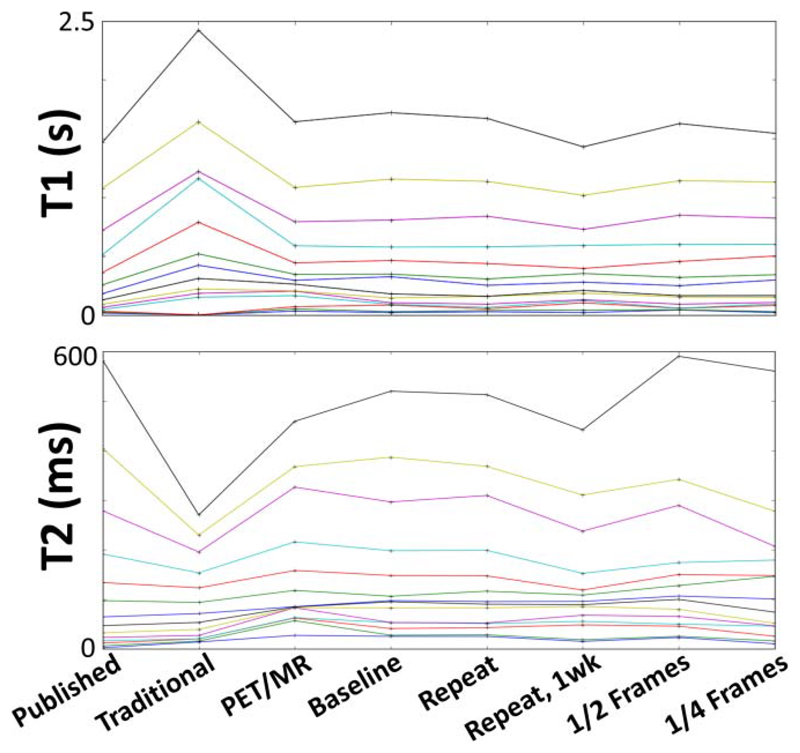
*T*_1_ and *T*_2_ values measured in the ISMRM/NIST phantom vials (of the *T*_1_-slice and *T*_2_-slice of the phantom, respectively). Each horizontal line corresponds to a single vial, which would be constant with repeatable and reproducible conditions. The labels indicate values obtained from: *Published* values in the NIST manual; *Traditional* quantification with standard *T*_2_ multiecho spin echo (ME*SE*) or *T*_1_ VFA mapping; using the *PET/MR*; on the clinical system (*Baseline*), *Repeat*ed after an hour and after 1 *week*, and using a reduced number of *Frames* (1/2 or 1/4) when compared with baseline (frames = 979).

The ISMRM/NIST phantom *T*_1_ and *T*_2_ values had <10% mean difference between the ratio of the baseline and the PET/MR, 1-h repeat, 1-week repeat, and reduced frame (1/2 and 1/4 length) maps (Fig. 2, Table II). The *T*_2_-MESE measurements underestimated the *T*_2_ by nearly 40% for the ~600 ms phantom vial but had <10% error between 20 and 160 ms. The *T*_1_-VFA measurements overestimated *T*_1_ values above 500 ms by > 60% and could not be fitted below 20 ms.

**Fig. 3:**
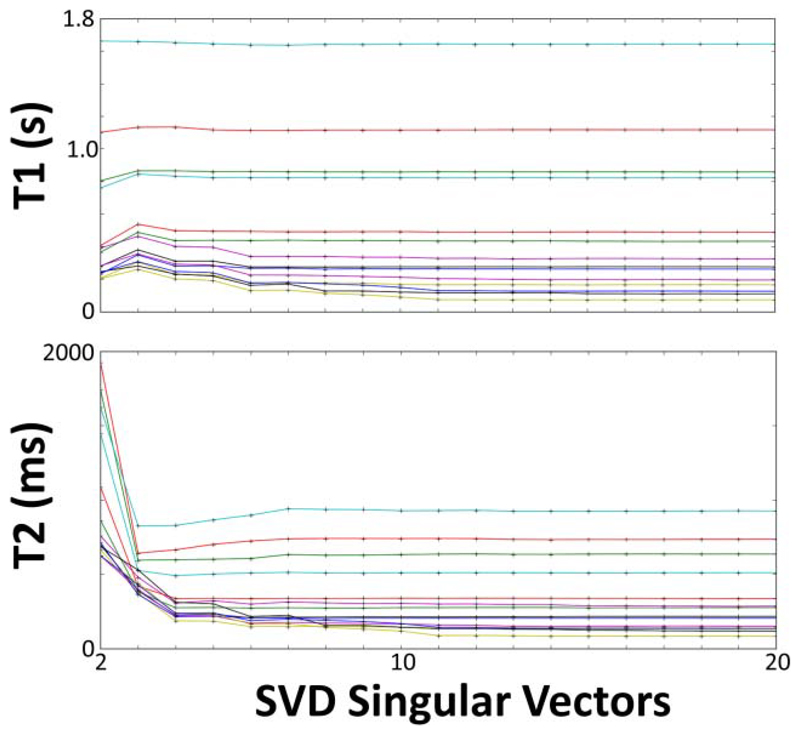
*T*_1_ and *T*_2_ values measured in the ISMRM/NIST phantom with the number of singular vectors for SVD compression (= rank) varied between 2 and 20 before matching. Each horizontal line represents a static *T*_1_ or *T*_2_ value. Both plots show values from vials in the *T*_2_-slice. Longer *T*_1_ and *T*_2_ values (>0.5 s) are accurate with rank > 3, while shorter relaxation values require rank > 16 to have less than 1% difference with rank 20.

The SVD rank affected *T*_2_ more than *T*_1_, with larger effects on lower *T*_1_ and *T*_2_ values (Fig. 3). A rank of one resulted in no *T*_1_ or *T*_2_ images. A rank of two enabled large *T*_1_ values (>0.8 s) to be matched within 10% of their rank 20 value, while lower *T*_1_ values required a rank of 11 or higher for similar differences. Similarly, *T*_2_ values >0.5 s were within 20% of their final value above rank 3, within 5% of their final value above rank 11, and within 1% above rank 16.

### B *In Vivo* 

Dictionary simulation was performed in 45 min. The abdominal images obtained with the 32-channel coil required 3.5 h for non-Cartesian reconstruction and matching, per subject.

**Fig. 4:**
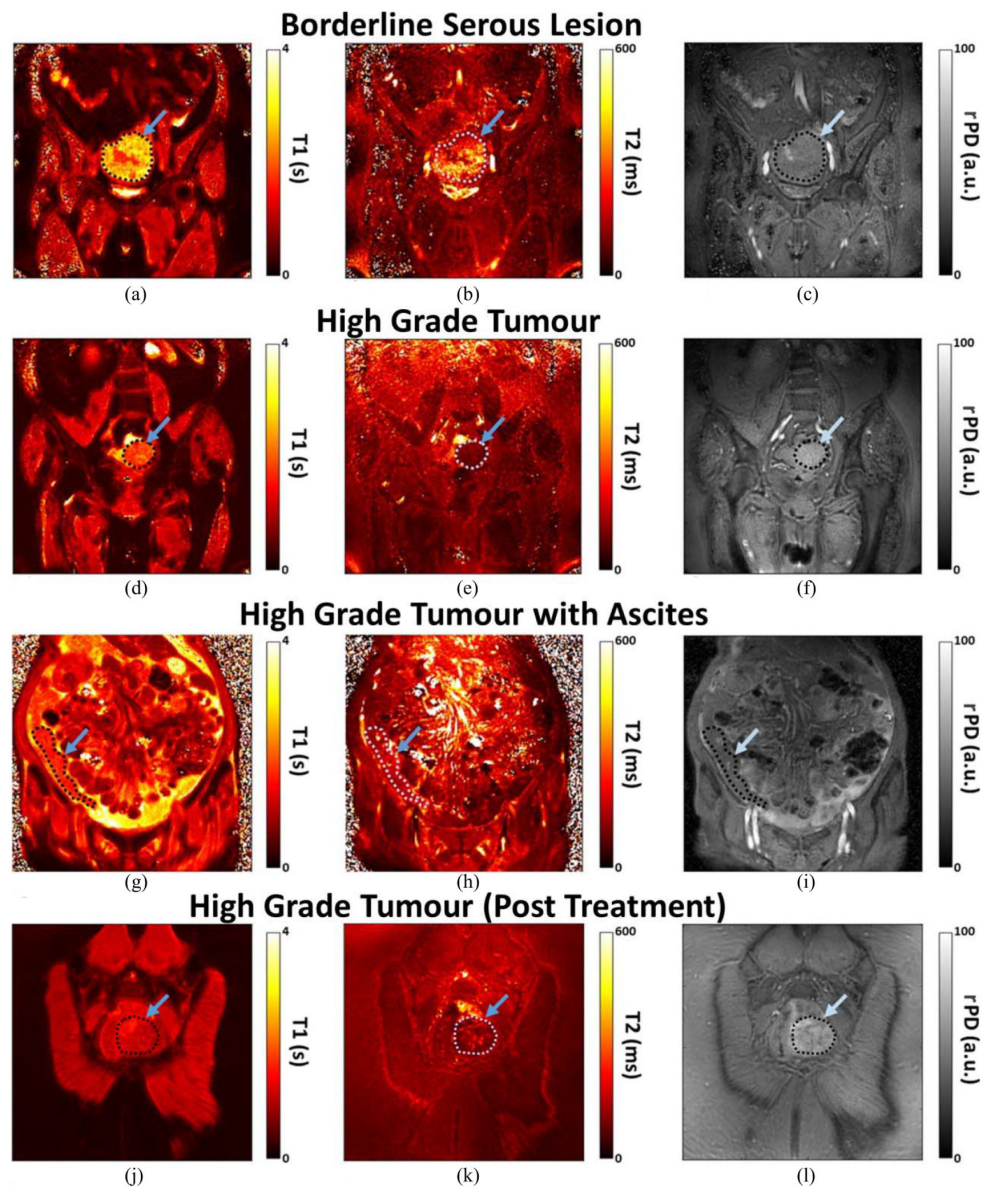
Coronal *T*_1_, *T*_2_, and PD quantitative maps of the four patients with (a)–(c) borderline serous and (d)–(l) high grade tumors. One of the HGSOC tumors had extensive ascites, and the HGSOC patient who had treatment had no histological evidence of remaining carcinoma (j)–(l). The *T*_2_ images (b), (e), (h), and (k) are shown with a maximum scale of 600 ms, although the dictionary was created up to values of 2.5 s.

The generated *T*_1_, *T*_2_, and rPD maps of the four patients are shown in Fig. 4. The mean *T*_1_ and *T*_2_ values and their standard deviations (SDs) estimated from the ROIs for each tumor type are listed in Table III.

The borderline serous ovarian tumor had a higher *T*_1_ value than the two HGSOC untreated lesions, which was discernable on the MRF maps. Mean *T*_1_ and *T*_2_ relaxation times of the borderline tumor were longer by ~20% and ~58%, respectively, when compared with the two pretreatment HGSOC tumors. The treated patient, with no evidence of remaining tumor, demonstrated lower *T*_1_ and *T*_2_ values from the other two HGSOC patients by 50%–150% for *T*_1_ and by 33%–50% for *T*_2_.

## IV Discussion

This proof-of-principle study has demonstrated the feasibility of using MRF for fast quantification of relaxation parameters in ovarian cancer for the first time. The quantitative MRF maps generated in this proof-of-concept work have demonstrated variations in *T*_1_ and *T*_2_ that could be assessed for further characterization of tumors in future studies.

### A Phantom

The baseline (979 frame) MRF demonstrated had <5% *T*_1_ differences from 1/2 and 1/4 frame experiments, which suggests that the parameter list can be reduced without loss of accuracy. A reduction in the list lengths would proportionally reduce the time required for acquisition and dictionary matching. Interestingly, the 1/2 and 1/4 frame lists were closer to published NIST value for *T*_2_, which, if confirmed in more experiments, could be a result of increasing the weight of signals affected by the initial inversion pulse before the list.

The MRF quantitative maps were closer to the accepted ISMRM/NIST values for a wider range of *T*_1_ and *T*_2_ values when compared to conventionally acquired quantitative maps in the ISMRM/NIST phantom (Fig. 2). The short TR (=1500 ms) was a likely source of error for vials with long *T*_1_ values during conventional *T*_2_ mapping. Nonuniform *B*1+ fields were a source of error for conventional *T*_1_ mapping. The MRF maps were not accurate for *T*_1_ or *T*_2_ values below 30 ms, which resulted in nearly similar values near 20 ms for four vials, despite the increased resolution of simulated *T*_1_ and *T*_2_ values. These short *T*_2_ ranges are important for fat quantitation, which has fast relaxation. Simulations are required to determine how to optimize for these values, which may require dramatically changing the TR or FA lists.

The compression of the images was performed at rank 16 for *in vivo* assessment because the phantom differences between rank 16 and higher rank were less than 1%. While increasing the rank would ideally result in higher precision, the compressed images beyond the first sixteen have noise-like appearances, suggesting negligible benefit with their inclusion. This noise-like appearance at high rank is likely due to an insufficient *in vivo* signal-to-noise ratio from the use of large abdominal coils, the presence of multiple tissue types, and motion artifact.

This is the first demonstration of ISMRM/NIST phantom MRF measurements on a PET/MR system. Previous work with MRF on a PET/MR has not assessed the phantom measurement accuracy [15].

### B *In Vivo* 

This feasibility study was a first abdominal MRF experiment on our system, so acquisition parameters were conservative. We used a conservative maximum gradient strength and slew rate to ensure sufficient signal-to-noise for accurate matching. *In vivo* mapping has challenges not present in phantom measurements, such as requiring a larger FOV, increasing receiver channel numbers, introducing artifact from respiratory motion, and increasing *B*_0_ and *B*_1_ field nonuniformity. Previous MRF of the abdomen was shown by Chen *et al*. [8], who used 2500 spiral arms (frames) for a single 5-mm thick slice, with the FOV = 44 cm, and with each frame rotated by 7.5°. Our *in vivo* work used 979 frames with golden angle spiral rotations and acquired 18-22 slices.

These results are promising in demonstrating the feasibility of MRF for quantitative pelvis and abdominal MRI. This feasibility study does not have sufficient numbers to demonstrate clinical benefit due to the limited patient sample size. Therefore, these preliminary results need to be validated in a larger cohort to assess the robustness of the results. This approach can be used to validate MRF as a fast and reliable alternative to the time-consuming gold-standard methods to acquire this data. Increased MRF resolution could be used to detect smaller tumor lesions in the future.

Ascites is difficult with traditional MRI methods because conductive ascitic fluid introduces standing waves that attenuate the radiofrequency fields, resulting in signal loss [16], [17]. Ascites-related loss is not visible in the maps of Fig. 4(g)–(i). Initial experiments with a reduced *T*_1_ and *T*_2_ parameter range [9] resulted in *T*_2_ values that were at the upper limit of the dictionary, so the dictionary range was extended (Table I) to map these compartments. Ascites map reconstruction did not otherwise present challenges different from the other subject maps.

Biological variation is visible on the MRF images [Fig. 4(a)–(c) and (j)–(l)], which is important for future work evaluating intratumoral heterogeneity: HGSOC is known to be very heterogeneous, which may be relevant for tumor progression and resistance to therapy [18]. Biological validation of image heterogeneity on histology will be important in future studies. Furthermore, MRF quantitative maps could be used in conjunction with radiomic measurements of tumor heterogeneity once these methods are validated.

Our nominal FOV(=38 cm) was chosen to image the whole abdomen, although was slightly smaller than the abdomen of three patients [Fig. 4(a)–(f) and (j)–(l)]. The maps of Fig. 4(d)–(f) and (j)–(l) do not appear to have image aliasing artifact that could be caused by a small FOV. Aliasing was reduced though the golden angle sampling pattern, which enabled a dense *k*-space that creates a large FOV, and reduces sensitivity to motion artifact [19]. Spiral acquisition regridding to a Cartesian *k*-space also enabled a large reconstructed FOV due to the interpolation of intermediate *k*-space values from coils with limited sensitivity regions [20], which acts similarly to other coil acceleration methods. Dictionary matching also reduced image aliasing, as the coils with the highest signal-to-noise contribute more than aliased, distant coils.

Respiratory artifact is not apparent on these images. Although the pelvis is subject to respiratory motion, these images were acquired with free-breathing during the 4-min scan. The pelvis is lower than other abdominal areas, and therefore is less sensitive to motion than the upper abdomen. MRF is relatively insensitive to motion because pattern matching occurs when a voxel is static for enough frames. It remains to be determined whether fewer frames reduces motion artifact due to faster acquisition speeds, or a larger number of frames reduces artifact due to increased signal averaging and samples that can be matched.

The original MRF paper by Ma *et al*. [4] used 32-coil channels, and acquired a single-slice matrix of 128 × 128 with 1000 frames. This paper acquired twice as much data per slice, while including more slices, and involved a lengthy 3–4 h reconstruction, which does not include the dictionary creation time. Due to the large number of simulated and imaging parameters, the data was reconstructed in chunks by matching groups of voxels and slices independently. A 3-frame sliding window [13] increased the reconstruction time and memory requirements. The 3-frame sliding window was necessary to reduce the variability on the *in vivo T*_2_ maps, which otherwise were very noisy when reconstructed with a single frame.

In addition to relaxation time measurements, the MRF methodology could be combined with other tissue property measurements, such as diffusion and perfusion, to gain more information for an improved classification of tumor masses. Combining metabolic information gained from uptake of the glucose analog, 2-deoxy-2-[^18^F]-fluoro-D-glucose (^18^F-FDG) on PET [21], [22] with the anatomical and functional information gained from MRI has a potential to improve the detection and characterization of tumors as well as assessment of treatment response.

## Figures and Tables

**Table I:** Ranges and Incremental Changes for *T*_1_ and *T*_2_

Parameter	Range (ms)	Increment (ms)
T1	10 – 400	10
	400 - 4000	20
T2	2 - 20	1
	20 - 400	2
	400 - 2500	20

**Table II:** Difference With Baseline (Mean ± StdDev)

	ΔT_1_/T_1,Baseline_ (%)	ΔT_2_/T_2,Baseline_ (%)
Published NIST	-28 ± 16	-38 ±34
T_1_-VFA or T_2_-MESE	25 ± 68	-39 ± 14
PET/MR	26 ± 38	17 ± 32
Baseline (979 Frames)	0 ± 0	0 ± 0
Repeat, 1 Hour	-4 ± 19	0.2 ± 3.9
Repeat, 1 Week	7 ± 19	-9 ± 19
½ (=489) Frames	3 ± 36	2 ± 11
¼ (=244) Frames	-1 ± 5	-18 ± 24

**Table III:** Mean Relaxation Values of Different Tumor Types

Tumour	T1 (ms)	T2 (ms)
Borderline serous	2465 ± 101	225 ± 34
HGSOC	1975 ± 191	94 ± 15
HGSOC with ascites	1621 ± 46	174 ± 26
HGSOC post treatment	1059 ± 55	116 ± 12
